# Automatic sleep–wake classification and Parkinson's disease recognition using multifeature fusion with support vector machine

**DOI:** 10.1111/cns.14708

**Published:** 2024-04-11

**Authors:** Yin Shen, Baogeng Huai, Xiaofeng Wang, Min Chen, Xiaoyue Shen, Min Han, Fei Su, Tao Xin

**Affiliations:** ^1^ Department of Neurosurgery The First Affiliated Hospital of Shandong First Medical University & Shandong Provincial Qianfoshan Hospital Jinan Shandong P. R. China; ^2^ Medical Science and Technology Innovation Center Shandong First Medical University and Shandong Academy of Medical Sciences Jinan Shandong P. R. China; ^3^ First Clinical Medical College, Shandong University of Traditional Chinese Medicine Jinan P. R. China; ^4^ Department of Radiology Shandong First Medical University & Shandong Academy of Medical Sciences Taian P. R. China; ^5^ Institute of Brain Science and Brain‐inspired Research, Shandong First Medical University & Shandong Academy of Medical Sciences Jinan Shandong P. R. China; ^6^ Shandong Institute of Brain Science and Brain‐inspired Research Jinan Shandong P. R. China

**Keywords:** corticomuscular coherence, electrocorticographic, electromyogram, Parkinson's disease, sleep–wake scoring, support vector machine

## Abstract

**Aims:**

Sleep disturbance is a prevalent nonmotor symptom of Parkinson's disease (PD), however, assessing sleep conditions is always time‐consuming and labor‐intensive. In this study, we performed an automatic sleep–wake state classification and early diagnosis of PD by analyzing the electrocorticography (ECoG) and electromyogram (EMG) signals of both normal and PD rats.

**Methods:**

The study utilized ECoG power, EMG amplitude, and corticomuscular coherence values extracted from normal and PD rats to construct sleep–wake scoring models based on the support vector machine algorithm. Subsequently, we incorporated feature values that could act as diagnostic markers for PD and then retrained the models, which could encompass the identification of vigilance states and the diagnosis of PD.

**Results:**

Features extracted from occipital ECoG signals were more suitable for constructing sleep–wake scoring models than those from frontal ECoG (average Cohen's kappa: 0.73 vs. 0.71). Additionally, after retraining, the new models demonstrated increased sensitivity to PD and accurately determined the sleep–wake states of rats (average Cohen's kappa: 0.79).

**Conclusion:**

This study accomplished the precise detection of substantia nigra lesions and the monitoring of sleep–wake states. The integration of circadian rhythm monitoring and disease state assessment has the potential to improve the efficacy of therapeutic strategies considerably.

## Graphical abstract

This study utilized electrocorticographic (ECoG) power, electromyogram (EMG) amplitude, and corticomuscular coherence (CMC) values to construct sleep–wake scoring and Parkinson's disease (PD) prediction models based on the support vector machine (SVM) algorithm. The models enable precise classification and identification of sleep–wake states and exhibit robust performance across different rats.

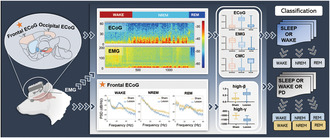

## 1 INTRODUCTION

Parkinson's disease (PD) is a widespread and progressive neurodegenerative disorder affecting individuals globally. The primary pathological change in PD is the selective degeneration of dopaminergic neurons in the substantia nigra pars compacta (SNpc), leading to decreased dopamine levels in the striatum and subsequent clinical symptoms.^1^ Although PD is mainly characterized by motor symptoms, nonmotor symptoms, such as sleep disturbances, are also significant and often appear earlier than motor symptoms.^2^ Sleep disorders in PD patients are commonly observed as daytime sleepiness, nocturnal insomnia, sleep fragmentation, and rapid eye movement sleep behavior disorder (RBD).^[^ ^3^, ^4^, ^5^ ^]^ Therefore, comprehensive circadian rhythm monitoring is crucial for early disease diagnosis and personalized treatment.

Extensive studies have been conducted to investigate sleep disturbances in PD. These studies used monitoring technologies such as electroencephalogram (EEG), electrocorticography (ECoG), electromyogram (EMG), and electrooculogram (EOG) to analyze the state of sleep and wakefulness (WAKE).^6^ With the development of digital signal processing technology, researchers have explored computer‐aided automatic sleep–wake scoring methods.^7^, ^8^, ^9^, ^10^, ^11^, ^12^, ^13^, ^14^ Petrovic et al. utilized EMG power and the δ/θ power ratio as features and employed a two‐cluster K‐means algorithm for three‐state classification.^7^ Crisler et al. selected various features from the parietal and frontal cortex, along with EMG, and utilized SVM for sleep classification in normal rats.^8^ Verma et al. focused on nonhuman primates rendered parkinsonian, using support vector machine (SVM) to classify daytime sleep–wake states based on different frequency band local field potentials (LFPs) recorded from the subthalamic nucleus (STN).^9^ Chen et al. employed SVM and decision tree algorithms on STN‐recorded LFPs powers across different frequency bands for sleep–wake classification in PD patients.^10^ These studies highlighted the overall superiority of SVM in the classification of neurophysiological data and underscored the significance of the β, α, and γ rhythms as classification features. Additionally, various classification methods, including the hidden Markov model,^12^ random forest,^13^ and deep learning networks,^14^ have been proposed to develop suitable sleep–wake scoring models. Among these, the SVM demonstrates enhanced efficiency in achieving real‐time automatic classification owing to its reduced training sample requirement and response time. Furthermore, the remarkable robustness makes SVM an optimal candidate for constructing the sleep–wake scoring model in our study.

The 6‐hydroxydopamine (6‐OHDA) lesioned rodent models are extensively employed in basic research due to their resemblance to PD patients.^15^ They display augmented daytime activity and more nocturnal sleep episodes during the night, resembling the characteristic features of nocturnal insomnia and daytime sleepiness, which are commonly observed in PD patients.^2^ These resemblances render the 6‐OHDA rats valuable for investigating the correlation between sleep architecture and disease progression.^16^ Gonzalez et al. used ECoG and EMG signals for sleep–wake classification in rats.^17^ ECoG signals are obtained by penetrating the skull with screws, which have considerable advantages over EEG, such as superior spatial resolution, spectral bandwidth, and signal‐noise ratio.^18^ The ECoG signals of rats in different states are similar to the EEG signals of humans, but there are also differences.^11^ For instance, elevated β waves (12.5–30 Hz) can be observed in the frontal motor cortex (M1) of PD rats,^19^, ^20^ which is similar to the EEG of PD patients. Meanwhile, raised θ waves (4.5–8 Hz) of hippocampal origin have been detected in the occipital cortex of rats during both WAKE and rapid eye movement (REM) sleep,^11^, ^21^ whereas elevated θ waves are only present during non‐rapid eye movement (NREM) sleep in humans. Hence, we conducted concurrent recordings of ECoG signals from the occipital and frontal lobes and further undertook a comparative analysis of signals originating from distinct brain regions across different rats to discern their contributions to the efficacy of sleep–wake scoring methods. The ultimate objective was to identify the optimal automatic sleep–wake scoring method for rats.

Furthermore, current common treatments for PD, such as levodopa medication and deep brain stimulation (DBS), aim to reduce the pathologically increased β oscillations in the STN and M1.^22^, ^23^ The β band activity is also a biomarker for adjusting closed‐loop DBS to manage side effects.^24^, ^25^ However, it should be noted that the β band power also decreases during NREM sleep, in addition to the effects of dopaminergic medication and DBS therapy.^26^ Therefore, it is necessary to identify additional biomarkers that can complement the role of β band power in closed‐loop modulation. This pursuit is envisioned to significantly amplify the efficacy of neuromodulation strategies through a cohesive integration of concurrent circadian rhythm monitoring and disease state assessment.^27^ In this study, the integration of the corticomuscular coherence (CMC) index into sleep–wake scoring emerged as a pivotal enhancement, notably boosting overall accuracy. Concurrently, we scrutinized the effectiveness of frontal and occipital ECoG in sleep–wake scoring, aiming to optimize the scoring method. Lastly, we introduced an innovative approach by fusing sleep–wake scoring with PD diagnosis, thereby broadening the scope of classifier applications. These findings contribute valuable theoretical insights into comprehensive neuromodulation strategies, holding the potential to reshape the landscape of early PD diagnosis and personalized treatment.

## 2 METHODS

### 2.1 Animals

In this study, 18 adult female Sprague–Dawley rats (weighing approximately 160–200 g) were obtained from Jinan Pengyue Experimental Animal Breeding Co., Ltd (Jinan, Shandong, China). All animals were housed in a room with free access to water, maintained at a constant temperature (21 ± 1°C), and under a natural light/dark cycle. The experimental protocol was approved by the Animal Experiment Ethics Committee of the First Affiliated Hospital of Shandong First Medical University [no. LCYJLL2022(009)].

### 2.2 6‐OHDA lesion and assessment

The rats were allocated randomly into two distinct groups: the lesion group (*n* = 9), where the rats received a unilateral lesion in the medial forebrain bundle (MFB) by 6‐OHDA, and the sham group (*n* = 9), where the rats were injected with 0.02% ascorbic acid in the MFB. Before the injection of 6‐OHDA 30 min, animals were pretreated with desipramine (5 mg/mL, i.p.) and pargyline (1 mg/mL, i.p.) to protect noradrenergic neurons and prevent premature decomposition of drugs.^28^ Rats were anesthetized with 1% pentobarbital and mounted in a stereotaxic frame. A vehicle solution (total volume 4 μL) containing 6‐OHDA (5 mg/mL) and 0.02% ascorbic acid in saline was injected into the left MFB according to the following stereotaxic coordinates relative to bregma and dura: AP = −4.40 mm, ML = −1.20 mm, DV = +7.80 mm.^29^ Leave the microsyringe in situ for a further 5 min. Control animals underwent identical surgical procedures but only received 0.02% ascorbic acid in the MFB.

The apomorphine‐induced (1 mg/mL, s.c.) contralateral rotational behavior was tested 7 days after the 6‐OHDA lesions to assess its effectiveness. Surgery was considered successful in animals that performed more than seven contralateral rotations to the 6‐OHDA treated site per 1 min and over 210 rotations within 30 min.^30^ Only nine rats with successful surgery were included in the lesioned group.

### 2.3 Electrode implantation and electrophysiological recordings

**FIGURE 1:**
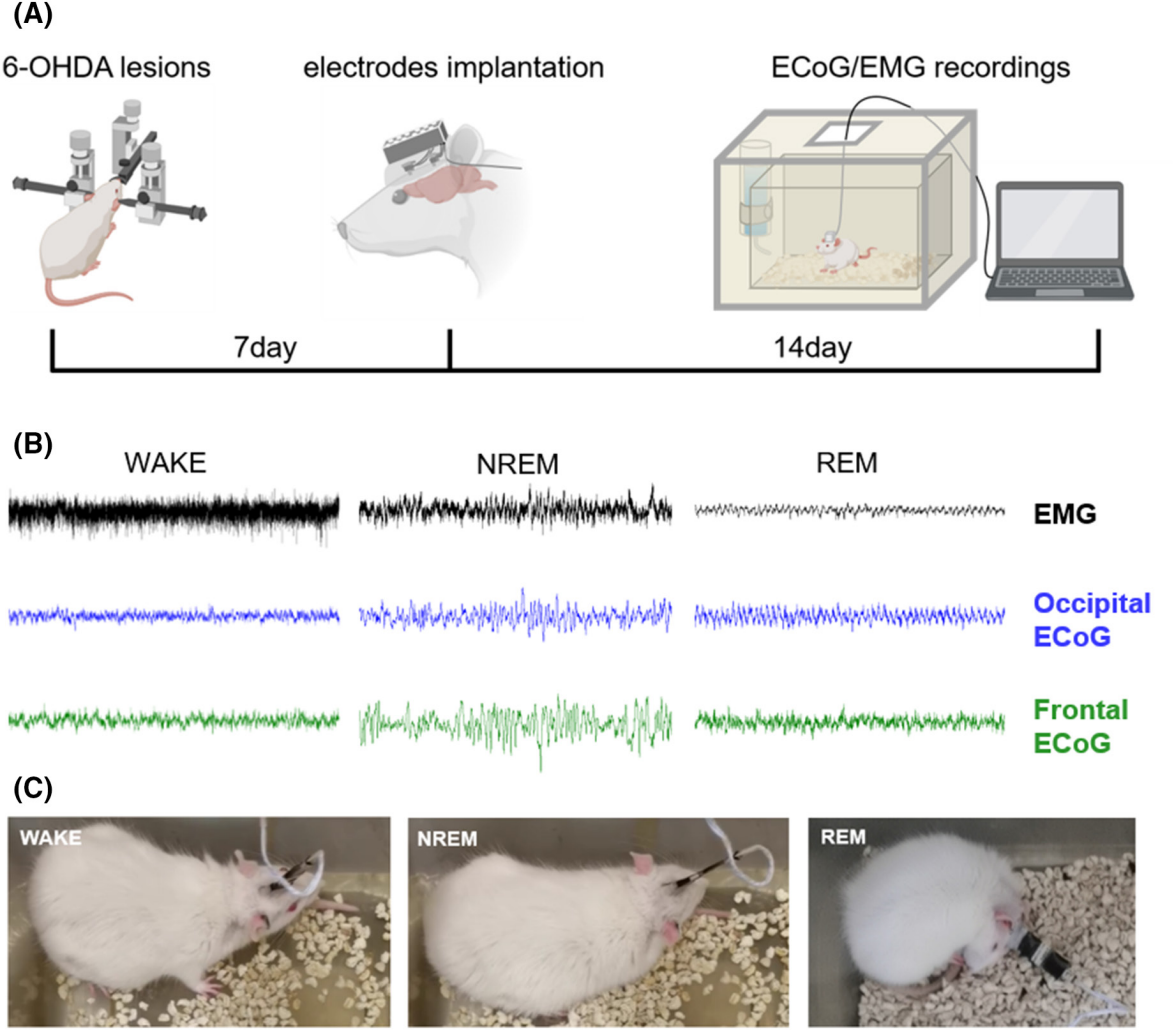
Experimental timeline and visual scoring criteria. (A) Schematic representation of the experimental design. (B) Typical waveform during WAKE, NREM sleep, and REM sleep states. (C) Typical behavior characteristics during the three states. Created with BioRender.com.

After 7 days of stereotactic injection surgery, the rats were implanted with ECoG and EMG electrodes for electrophysiological recordings (Figure 1A). We implanted four epidural stainless‐steel screw electrodes for the recording of ECoG activity from the bilateral frontal (AP = +2.00 mm, ML = ±2.00 mm, DV = +1.00 mm) and occipital (AP = −6.00 mm, ML = ±2.00 mm, DV = +1.00 mm) regions of the cortex.^29^, ^31^ (details in the supplemental files).

The electrophysiological recordings were performed 14 days after the electrode implantation surgery.^7^ Each rat was placed in the Faraday cage for 1 day to adapt to the environment before recording. The electrophysiological recording was performed for 2 h in the afternoon, containing three states of the sleep–wake cycle. We sampled the ECoG and EMG signals at 1000 Hz, then amplified and processed the signals using OmniPlex (Plexon, USA). The sleep–wake analyses were done by the Offline Sorter (Plexon, USA), NeuroExplorer (Nex Technologies, USA), and MATLAB (The Mathworks, USA).

### 2.4 Visual scoring criteria

We adopted visual scoring criteria established in previous studies to assess the electrophysiological recordings.^11^, ^32^, ^33^ Each sleep recording from the cohort of 18 rats underwent meticulous annotation, employing a dual‐expert approach for visual scoring in 10‐s segments, with each expert specializing in different aspects of the data. The first expert focused on discerning sleep states based on the observed behavior of the rats as shown in Figure 1C,^32^ whereas the second expert concentrated on the analysis of electrophysiological recordings, specifically ECoG and EMG waveforms as shown in Figure 1B.^11^, ^33^ During the WAKE state, the cortex region displays low‐amplitude and fast ECoG activity while exhibiting high‐amplitude EMG activity. During both NREM and REM sleep states, the rats displayed behavioral immobility and exhibited low EMG amplitudes. However, NREM sleep is associated with high‐amplitude and fast ECoG activity, whereas the ECoG waveform during REM sleep is similar to that observed during WAKE. Following independent assessments of sleep–wake states, the two experts collaboratively reviewed their findings to achieve a consensus.

### 2.5 Feature selection of neurophysiological signals

By analyzing neurophysiological signals derived from the cortex and muscle, we aimed to discern the significant characteristics inherent in ECoG and EMG signals across distinct frequency domains, thereby identifying the most informative features for accurate classification.

We used notch filtering to filter the 50 Hz frequency band power line artifacts from the analysis. The 2‐h data were divided into 10‐s segments. A Fast Fourier Transform (FFT) analysis was performed to evaluate the ECoG power across the seven conventional bandwidths (δ, 0.5–4 Hz; θ, 4.5–8 Hz; α, 8.5–12 Hz; low‐β, 12.5–20 Hz; high‐β, 20.5–30 Hz; low‐γ, 30.5–40 Hz; high‐γ, 40.5–60 Hz).^11^ For each frequency band, the mean power was treated as a feature of the 10‐s segment. The EMG signal was filtered within the range of 20–200 Hz,^34^ and the mean absolute amplitude of the filtered EMG was also calculated as a feature of the 10‐s segment. The CMC values were determined to measure the linear synchronization between ECoG and EMG signals across the seven frequency bands,^35^ with values ranging between 0 and 1, which provided insights into the degree of coherence between the two signals.^36^

A normalization procedure was employed to normalize the indices on a scale of 0–1 to reduce errors.^11^ Furthermore, an additional comparison was performed using the unnormalized power of the frontal ECoG signal to determine an appropriate index for PD classification.

Following the initial data assessment, the normality of the data was evaluated using the Kolmogorov–Smirnov test. Subsequent analysis was conducted based on the distribution of the data. If the data adhered to a normal distribution, the t‐test and analysis of variance (ANOVA) were performed to examine group differences. Conversely, if the data deviated from a normal distribution, nonparametric tests such as the Mann–Whitney U test and the Kruskal–Wallis test were employed to evaluate group distinctions. GraphPad Prism software (GraphPad Software, USA) was utilized for statistical analysis, and the outcomes were visually presented using scatter plots.

### 2.6 Model construction and application

To evaluate the applicability of the sleep–wake classification models across different rat groups, we randomly selected the features from 8 rats in the sham group (Sham train) and 8 rats in the lesion group (Lesion train) to train the SVM models, respectively. Subsequently, the remaining sham record (Sham test) and lesion record (Lesion test) were considered as two test data points for cross‐validation of the models. In the present investigation, a kernelized SVM classification model was utilized, incorporating the penalty factor C, with the radial basis function (RBF) serving as the kernel function. The parameter C in the SVM model reflects the influence of samples within the margin on the overall error. The optimization of C spanned the range between 1 and 2^7^. Additionally, concerning the RBF kernel function in the SVM model, the parameter γ underwent fine‐tuning within the interval of 2^−7^ to 1.^10^

**FIGURE 2:**
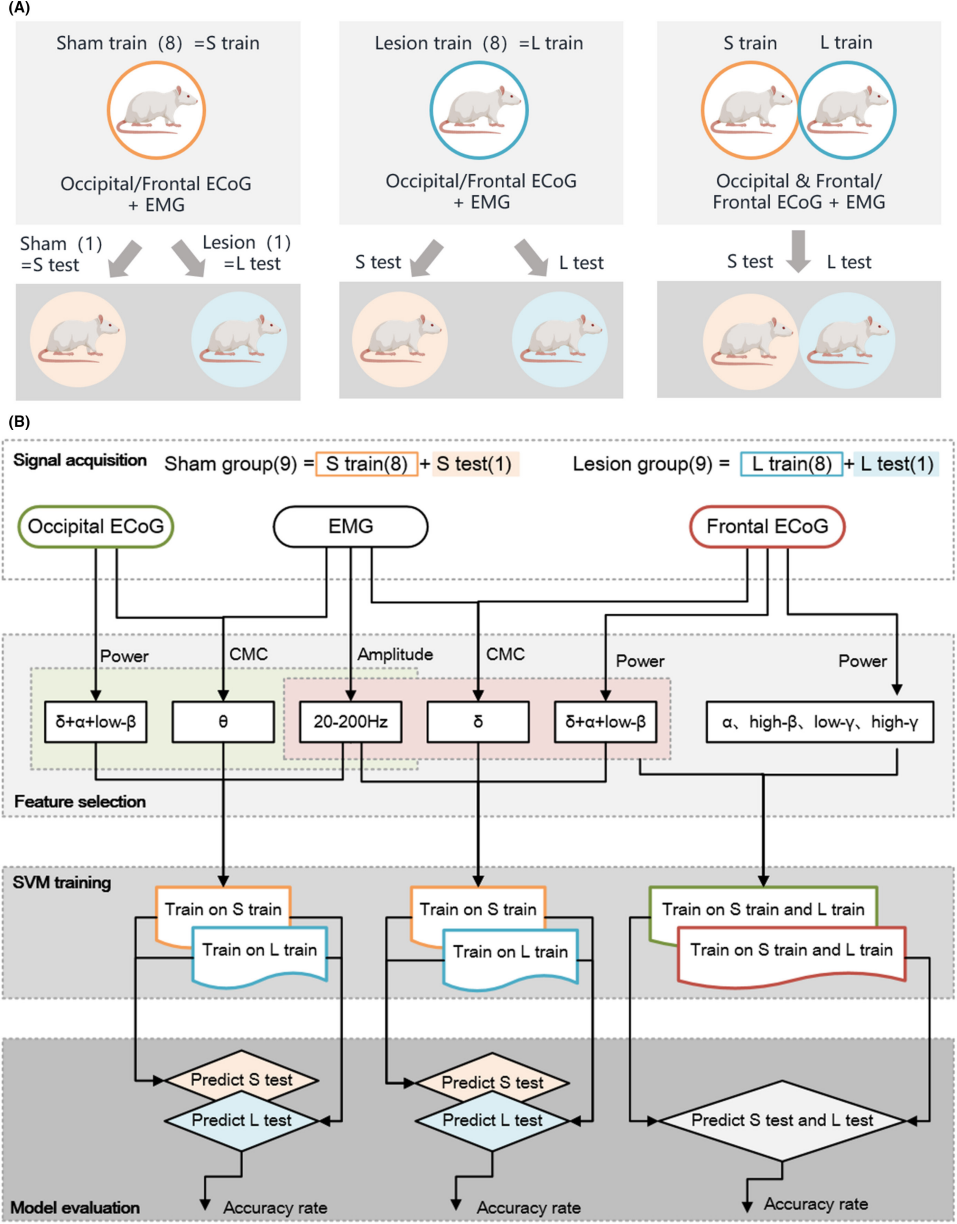
The automatic sleep–wake classification and PD prediction program flow chart. (A) Categorization of experimental animals and the determination of sets for training and testing. The S train and the L train correspond to data from eight rats in the Sham and Lesion groups, respectively. The S test and the L test correspond to data from the remaining rats in the Sham and Lesion groups, respectively. (B) The average power of ECoG, the mean amplitude of EMG, and the CMC values are extracted from the training data (S train and L train). The appropriate features are selected and input into the SVM training models. Then, we test the remaining data (S test and L test). The classification results were compared with the visual scoring outcome to evaluate the performance of the models. Created with BioRender.com.

The Sham dataset contained 6480 10‐s segments labeled by the sleep experts with data encompassing WAKE: 1548, NREM: 4164, REM: 768. Similarly, the Lesion dataset contained 6521 10‐s segments labeled by the sleep experts with data encompassing WAKE: 2047, NREM: 3866, REM: 608. The Sham test and Lesion test contained approximately 720 10‐s segments, respectively. For comparison, we used ECoG from occipital or frontal cortex regions in the Sham train and Lesion train for feature extraction and model training to construct four different sleep–wake classification models (Figure 2).

Building upon this foundation, we conducted a comparative analysis of ECoG power in the frontal region between the two groups in three states. Then, we chose the PD characteristic signals from the frontal region to train a PD identification scoring model. The rats were systematically grouped into pairs, with the data from eight pairs utilized for model construction. The efficacy of the model was assessed by testing the remaining pair of rats. We additionally evaluated the performance of the classification model by plotting the receiver operating characteristic (ROC) curve and calculating the area under the curve (AUC).

Finally, we utilized the ECoG and EMG signals for sleep–wake staging combined with the PD characteristic signals from the frontal region in the Sham train and Lesion train to train the PD recognition and sleep–wake scoring models. A total of two models were generated and tested on the remaining two rats in the two groups to determine the more appropriate feature combination method. By incorporating the previously selected features and expanding the functionality of the model, we achieved simultaneous detection of sleep–wake states and the likelihood of PD in rats.

Confusion matrixes were constructed to calculate the precision, sensitivity, specificity, overall accuracy, and Cohen's kappa for each method to evaluate the performance of different classifiers. (details in the Files S1).

### 2.7 Tyrosine hydroxylases immunocytochemistry

The lesions were confirmed and assessed for quantitative analysis at the end of all the recordings. To assess the degree of dopaminergic degeneration, we analyzed sections of the SNpc and striatum obtained from rats that received 6‐OHDA injections. Immunocytochemical staining targeting tyrosine hydroxylase (TH) was performed on these sections (details in the Files S1).

## 3 RESULTS

### 3.1 Signal features in the frequency domain during three states

**FIGURE 3:**
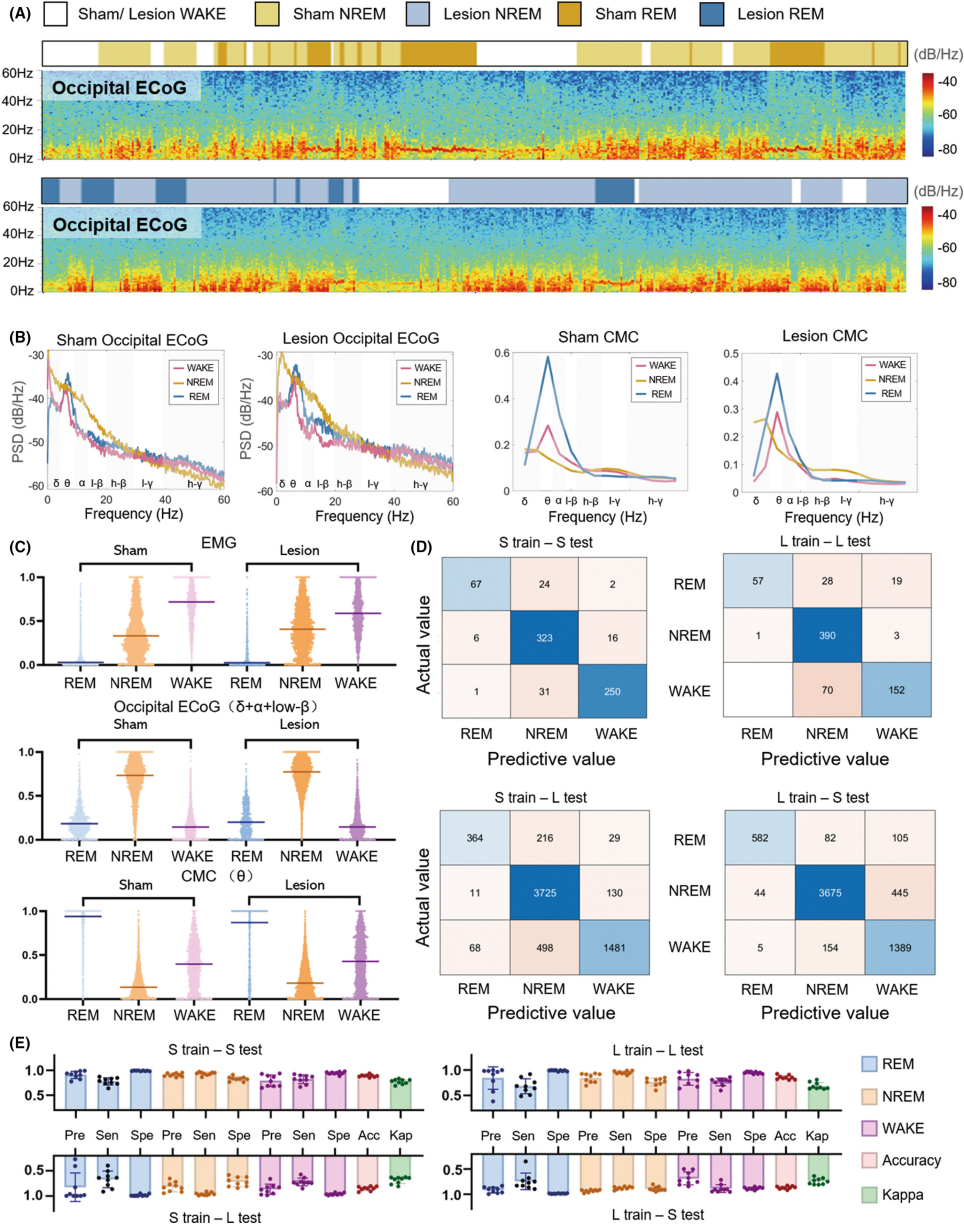
Statistical analysis of the EMG, Occipital ECoG, and CMC in the train data, with the confusion matrix for the test data. (A) The spectrogram of Occipital ECoG during WAKE, NREM sleep, and REM sleep states in the Sham and Lesion groups. (B) The mean Occipital ECoG power and CMC values in the two groups. (C) The distribution of EMG amplitude differs in the three states (Kruskal–Wallis test and Dunn's multiple comparisons post hoc test, *p* < 0.0001). The ‘δ + α + low‐β’ frequency band Occipital ECoG power increase is statistically significant during NREM sleep (Kruskal–Wallis test and Dunn's multiple comparisons post hoc test, *p* < 0.0001). The CMC of the θ band is significantly different among the three states (Kruskal–Wallis test and Dunn's multiple comparisons post hoc test). With the exception of the *p*‐value for ECoG power between WAKE and REM sleep in the sham group (0.0509) and the *p*‐value for ECoG power between WAKE and REM sleep in the lesion group (0.1073), all other *p*‐values were below 0.0001. (D) Four confusion matrices depict the outcomes of cross‐validation, where a model trained on Occipital ECoG and EMG data from one group of rats was tested on both this group and another, and vice versa. (E) Performance of sleep–wake scoring method using occipital ECoG.

**FIGURE 4:**
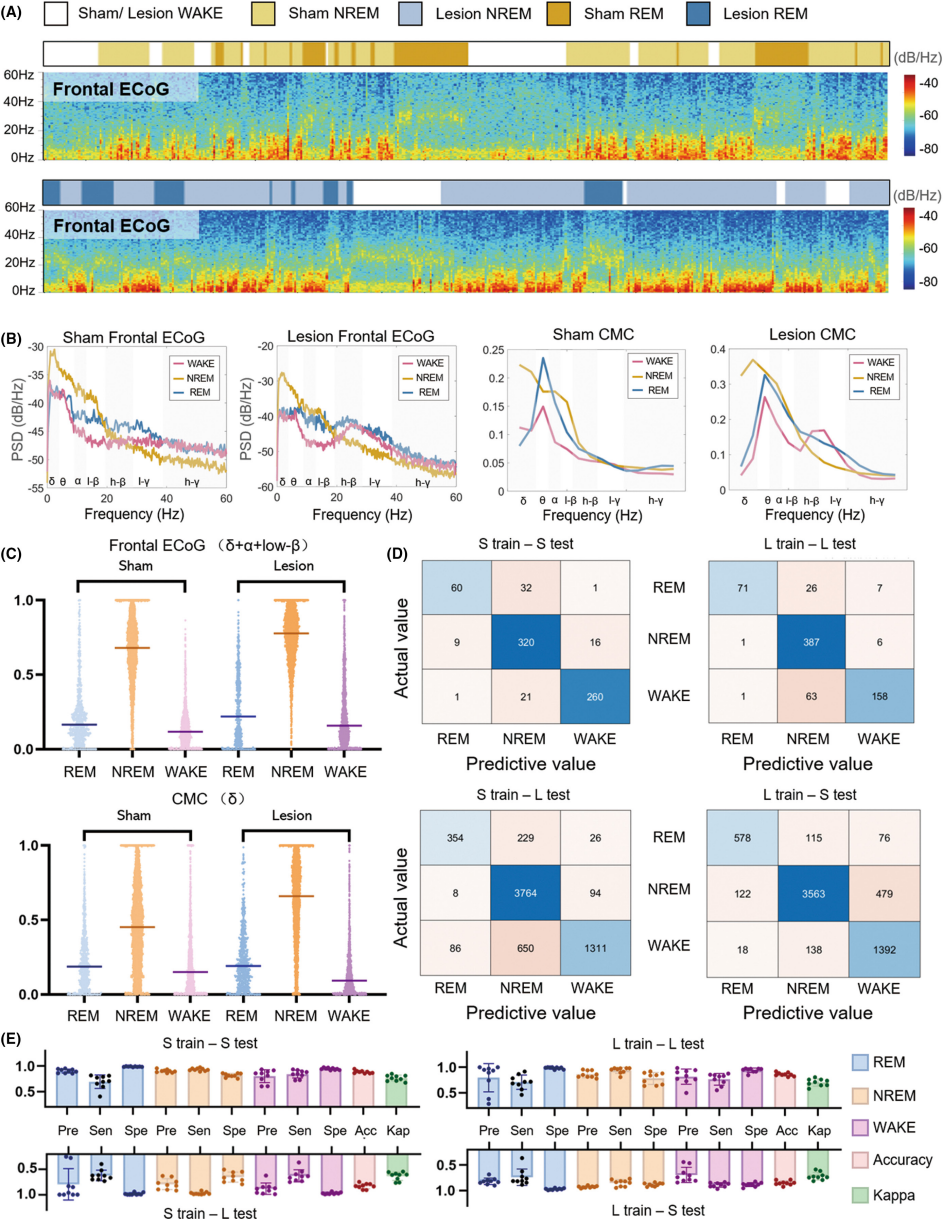
Statistical analysis of the frontal ECoG and CMC in the train data, with the confusion matrix for the test data. (A) The spectrogram of frontal ECoG during WAKE, NREM sleep, and REM sleep states in the Sham and Lesion groups. (B) The mean frontal ECoG power and CMC in the two groups. (C) The frontal ECoG power of the “δ + α + low‐β” frequency band and the CMC of the δ band increase statistically significantly during NREM sleep in the occipital lobe (Kruskal–Wallis test and Dunn's multiple comparisons post hoc test). With the exception of the *p*‐value for ECoG power between WAKE and REM sleep in the lesion group (0.4460) and the *p*‐value for CMC values between WAKE and REM sleep in the sham group (0.2076), all other *p*‐values were below 0.0001. (D) Four confusion matrices depict the outcomes of cross‐validation, where a model trained on frontal ECoG and EMG data from one group of rats was tested on both this group and another and vice versa. (E) Performance of sleep–wake scoring method using frontal ECoG.

Our findings demonstrated distinct patterns in the neurophysiological signals during different vigilance states (Figures 3A and 4A). Specifically, the EMG amplitude gradually decreased from WAKE to REM sleep (Figure 3B). At the same time, ECoG power displayed an increase in the NREM sleep state within the δ, α, and low‐β band compared with the WAKE and REM sleep states in both groups (Figures 3B and 4B). Notably, the coherence value between occipital ECoG and EMG signals in the θ band exhibited a pronounced peak during REM sleep while reaching its lowest point during NREM sleep (Figure 3B). Furthermore, the coherence value between frontal ECoG and EMG signals in the δ band peaked during NREM sleep (Figure 4B). These results highlight the dynamic interplay between vigilance states and the corresponding alterations in EMG amplitude, ECoG power, and CMC values within distinct frequency bands.

### 3.2 Alterations in frontal ECoG power between different frequency bands in two groups

**FIGURE 5:**
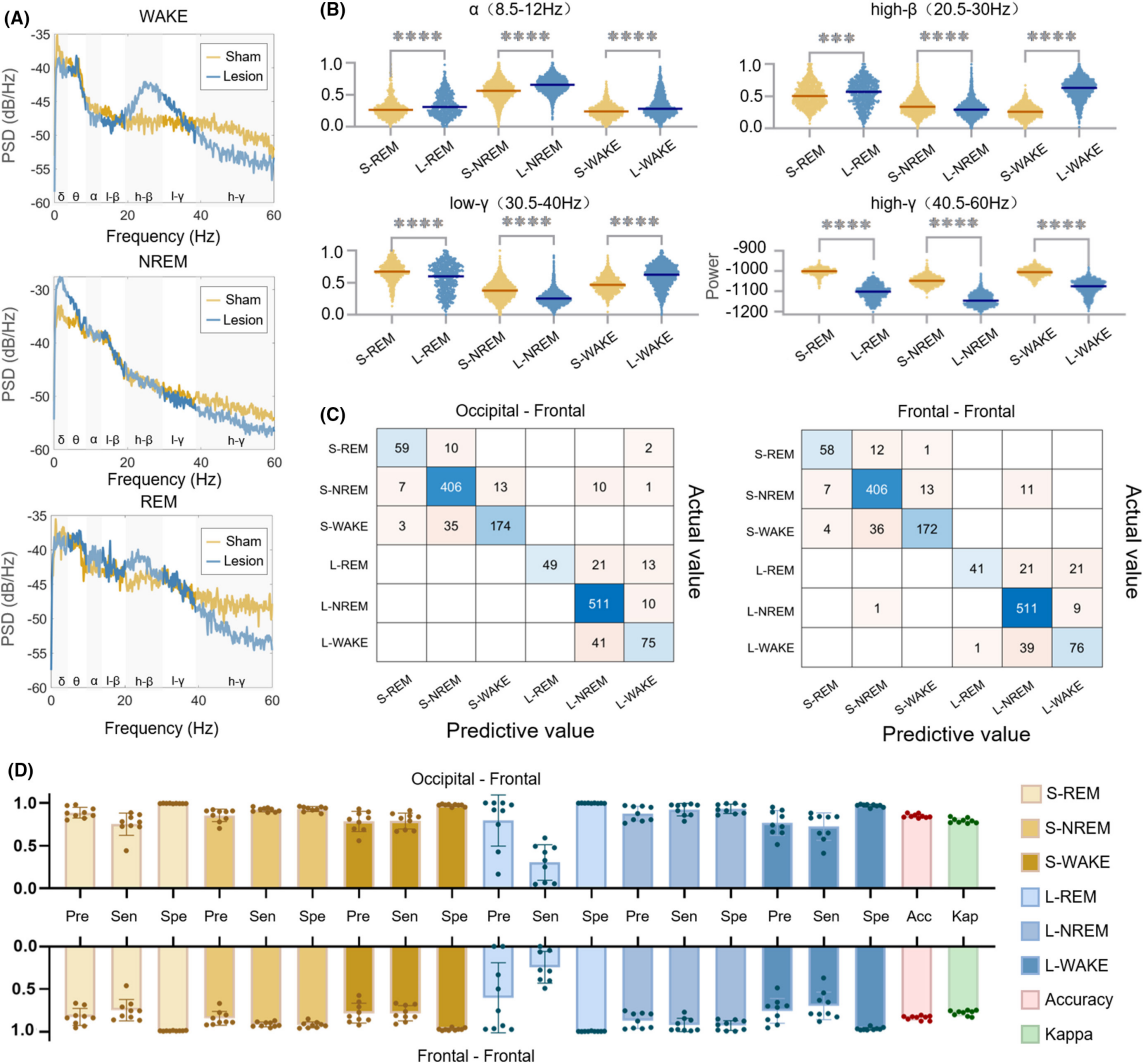
Statistical analysis of frontal ECoG power in two groups and the confusion matrix for the test data. (A) Power spectral density analysis revealed increased spectral power in the high‐β frequency range during WAKE and REM sleep in the lesion group compared with the sham group. (B) Comparative analysis of normalized power in the α (8.5–12 Hz), high‐β (20.5–30 Hz), low‐γ (30.5‐40 Hz) bands, and unnormalized power in the high‐γ (40.5–60 Hz) band demonstrated significant differences between the two groups during all three states (Mann–Whitney U test, *p* < 0.001). (C) Two confusion matrices show the classification results of models trained by Frontal ECoG and Occipital‐Frontal ECoG combined with EMG. (D) Performance of sleep–wake scoring and PD prediction models.

We further quantified the ECoG power from the frontal regions of the cortex by plotting the power spectral density and scatter plots (Figure 5). We observed increased power in the 20–60 Hz frequency band during NREM sleep. During WAKE and REM, there was an increase in the 20–40 band power as a result of SNpc lesions (Figure 5A). Upon conducting a Mann–Whitney U test on the power values between these two groups, significant statistical differences were observed in the α, high‐β, and low‐γ power after normalization and the high‐γ power before normalization across the three periods (Figure 5B).

### 3.3 Classification of WAKE, NREM sleep, and REM sleep

Following the features above, SVM models were employed to classify the three states in different rats. The classification and prediction processes are depicted in Figure 2. The assessment of sleep–wake scoring was conducted by comparing the visual scoring with the automated scoring for the remaining rats, as illustrated in Figures 3C and 4C. The confusion matrices illustrated the results of the testing process (Figures 3D and 4D). The overall accuracy rate ranged between 80% and 90% (Tables S1–S4). The highest accuracy was achieved when the model was trained by the Sham train of the occipital ECoG and tested by the Sham test data (Figures 3E and 4E), at which time the Cohen's kappa value was 0.77 ± 0.05 (Table S1).

### 3.4 Performance of the PD diagnostic classifier

In this segment, we evaluated our classifier based on an analysis of four selected features derived from the frontal lobe across three states in two groups (Figure 5B). The performance of this classifier in diagnosing PD has yielded outstanding results. The diagnostic accuracy reached an impressive 96.71% ± 3.07%, and the kappa value stood at 0.93 ± 0.06 (Table S5). Furthermore, the AUC value was 0.99, confirming the high discriminatory power of the model (Figure S1).

### 3.5 Effect of the sleep–wake scoring and PD diagnostic models

While selecting sleep–wake classification indices derived from distinct ECoG sources alongside shared EMG and PD classification indices for model training (Figure 5C), our investigation revealed an overall accuracy and kappa value of approximately 84% and 0.79, respectively (Figure 5D; Tables S6 and S7). The integrated classifier exhibited high specificity in classifying PD during REM sleep but showed considerable variability in precision, coupled with relatively low sensitivity. The presence of RBD in PD is likely to induce irregularities in electrophysiological signals, thereby contributing to the observed reduction in sensitivity during REM sleep classification.^37^ A slightly greater level of overall accuracy was noted when conducting classification through a combination of occipital and frontal ECoG, as opposed to relying solely on frontal ECoG (84.87% ± 2.22% vs. 83.90% ± 2.50%).

### 3.6 Expression of TH in SNpc and striatum

**FIGURE 6:**
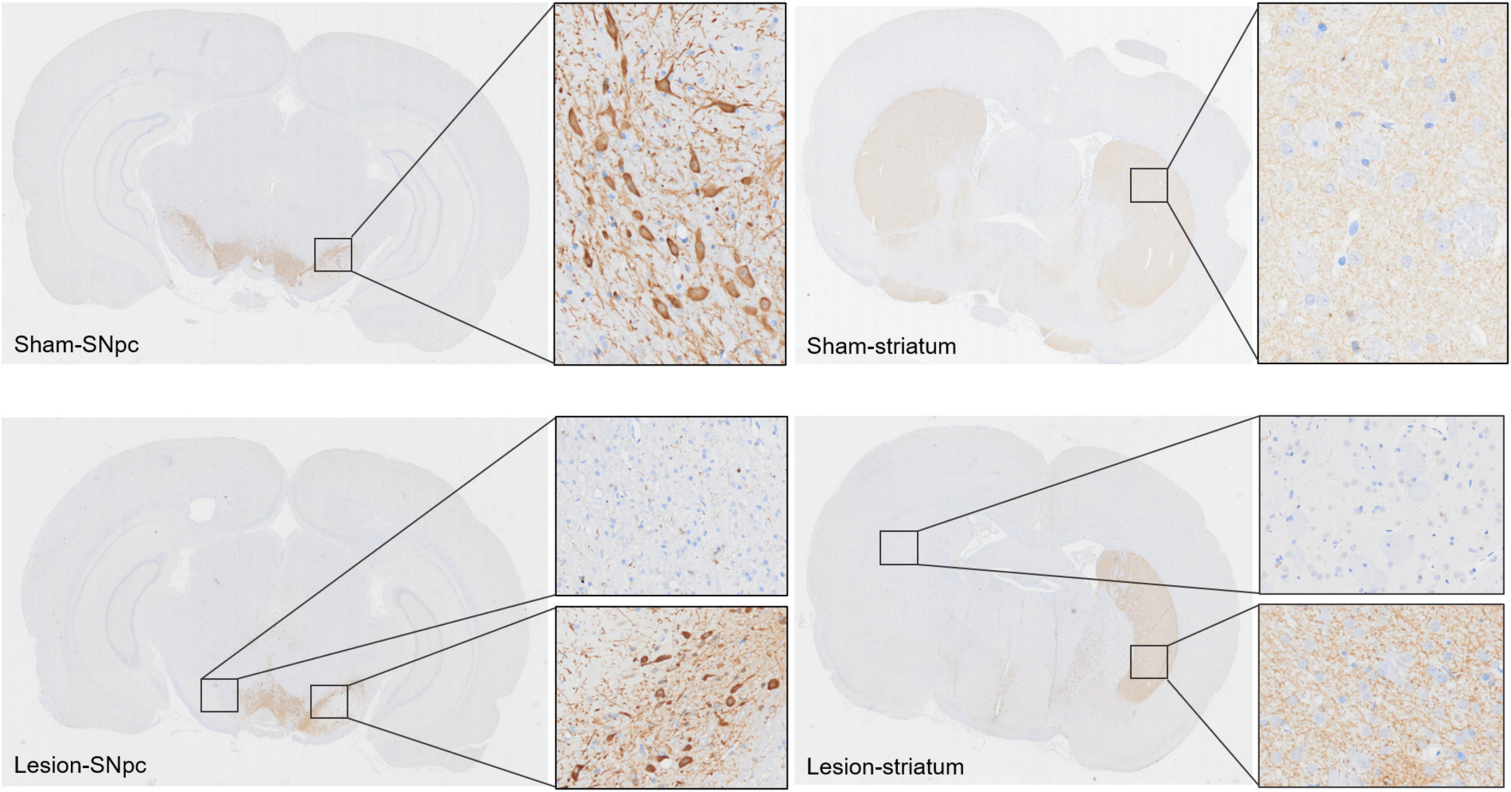
Histological identification of the dopaminergic neuronal loss. Microphotographs of the SNpc and the striatum TH immunohistochemistry in the Sham and Lesion groups.

In this study, the dopamine content in the brain was indirectly assessed by quantifying the content of TH. Immunohistochemical staining of TH was conducted on the SNpc and striatum of the rats. Compared with the sham group, the lesion group exhibited significantly reduced TH immunoreactivity in unilateral SNpc and striatum due to the loss of unilateral dopaminergic terminals (Figure 6). In the lesion group, the number of TH‐positive neurons was significantly reduced on the 6‐OHDA lesioned side compared with that on the other side.

## 4 DISCUSSION

Sleep disturbances have been found in more than half of PD patients, often occurring as an early manifestation of neurodegeneration.^38^ This research employed a fusion of ECoG and EMG signals in conjunction with SVM to categorize sleep–wake states. Our approach demonstrated high average accuracy (86.36%) in discriminating sleep–wake states between different rats. During the data acquisition, we observed distinctive features in the ECoG signals of distinct brain regions across diverse frequency ranges. During WAKE, our study found high levels of EMG activity accompanied by θ oscillations in the occipital ECoG, consistent with previous studies.^39^ It is worth noting that during REM sleep, the peak in θ activity is localized in the occipital cortex rather than in the frontal cortex. This observation could potentially be attributed to the sensitivity of occipital ECoG in capturing hippocampal θ activity.^40^, ^41^ Furthermore, an increase in power in the 8.5–20 Hz frequency bands in frontal and occipital cortical areas might indicate the emergence of sleep spindles.^42^, ^43^ In future studies, the integration of waveform analysis into power monitoring, complemented by the utilization of deep learning for feature extraction, holds the potential to enhance the detailed characterization of NREM and REM sleep states.

Additionally, we investigated the CMC, which reflects the interaction between cortical regions and muscle tissue.^44^ Our observations align with the finding by Ciric et al. that the coherence of the motor cortex to the muscle is higher in the θ band during WAKE and REM sleep and higher in the δ and β bands during NREM sleep.^45^

It has been established that dopamine depletion is associated with changes in the frontal M1 ECoG frequency, particularly in the high‐β and low‐γ ranges.^20^, ^46^, ^47^, ^48^ Our findings revealed that impairment of the SNpc leads to increased activity in the high‐β and low‐γ bands during WAKE and REM sleep. At the same time, we found that the CMC index also increased in these two frequency bands during WAKE, possibly due to the enhanced functional connectivity of the corticospinal pathway, leading to the downward conduction of this pathological oscillation. Moreover, the power of the β band decreased during NREM sleep, and the power of the high‐γ band decreased during all three periods. The present findings agree with those of previous studies that reported a negative correlation between β and high‐γ frequencies,^9^, ^48^ which may be related to the regulation of dopamine receptors. Furthermore, abnormalities in α oscillations in the frontal cortex during WAKE and NREM sleep may result from abnormal transmission of neural signals within cortico‐basal ganglia circuits.^49^ However, the mechanism of power changes in different frequency bands of cortical neurons remains unclear. It is anticipated that applying novel imaging techniques and multichannel EEG recordings will provide a better understanding of the intricate interplay between typical oscillatory and synchronized activities within the cortico‐basal ganglia motor circuit and muscle.^50^, ^51^

Current research suggests that excessive β band activity in the motor circuit may contribute to motor symptoms of PD.^52^, ^53^ High‐frequency electrical stimulation of the STN, located in the basal ganglia, can suppress β oscillations, which are associated with bradykinesia and rigidity.^54^ Using β oscillations as a biomarker for closed‐loop DBS has substantial advantages in improving motor function compared with traditional continuous or random stimulation^51^, ^55^, ^56^ while also greatly reducing stimulation time, significantly extending the service life of pulse generators. There is some evidence that STN stimulation may improve sleep–wake functions,^57^, ^58^, ^59^ but reductions in β oscillations during sleep may negatively affect therapeutic efficacy.^3^ As a result, many studies are now focusing on changes in brain electrical activity during sleep in PD patients to optimize closed‐loop DBS control programs for better therapeutic effects.^26^, ^60^ We also screened new biomarkers for assisting β oscillations in the diagnosis of PD by comparing ECoG differences in different vigilance states. We integrated these with the indices used for sleep–wake scoring to retrain the models. The average accuracy (84.39%) of the two retrained models for distinguishing vigilance states and identifying PD is very high. In the future, we will integrate our automated scoring algorithm into closed‐loop DBS stimulation procedures. We hope that the automatic classification model designed in our study can help identify the circadian rhythm of PD and improve closed‐loop DBS treatment for PD patients.

## 5 CONCLUSION

In this study, we have developed and validated a novel automated system for sleep–wake scoring and PD prediction in rats. The proposed method leverages multiple features extracted from ECoG and EMG signals. Using power features extracted from occipital and frontal ECoG signals, the average combined accuracy of the sleep–wake scoring method was 86.97% and 85.76%, respectively. The overall accuracy of simultaneous PD prediction was about 84.39%. Furthermore, the algorithms developed in this research exhibit promising potential for future integration into adaptable, wireless, and portable DBS monitoring systems.

## CONFLICT OF INTEREST STATEMENT

The authors affirm that there are no apparent conflicting financial interests or personal relationships that may have influenced the findings presented in this study.

## Supporting information


Data S1.

## Data Availability

The corresponding authors can provide the codes and data upon a reasonable request.
