# Supplementary material for: Automatic sleep–wake classification and Parkinson's disease recognition using multifeature fusion with support vector machine
Source: CNS Neurosci Ther. 2024 Apr 11;30(4):e14708. doi: 10.1111/cns.14708 (PMC11007385; doi:10.1111/cns.14708)
Supplement: Supplementary file 1 — Data S1. [file CNS-30-e14708-s001.docx]

**Electrode implantation and electrophysiological recordings**

Given the anticipated elevation of β activity in the frontal ECoG of the lesioned hemisphere, we choose the frontal ECoG from the lesioned side to analyze. On the other hand, the occipital ECoG exhibited no discernible disparity between the bilateral hemispheres, and we randomly selected one side of occipital ECoG data for further analysis. In addition, ground and reference electrodes were placed at the nasal bone and the bone above the cerebellum. Bilateral wire electrodes were implanted into the dorsal nuchal musculature to assess skeletal EMG. The electrode plug was secured to the skull with acrylic dental cement.

**Model construction and application**

Confusion matrixes were constructed to evaluate the concordance among different methods, which typically comprise the true positive (TP), true negative (TN), false positive (FP), and false negative (FN) classifications. The overall accuracy (P) and the expected classification accuracy (Pe) are computed. Subsequently, the precision, sensitivity, specificity, overall accuracy, and Cohen's kappa are derived for each method. Definitions of all indexes are provided below.

$$precision= \frac{TP}{TP+FP}\times100\%$$

$$sensitivity= \frac{TP}{TP+FN}\times100\%$$

$$specificity= \frac{TN}{TN+FP}\times100\%$$

$$overall accuracy=P= \frac{TP+TN}{TP+TN+FP+FN}\times100\%$$

$$Cohen's kappa= \frac{P-Pe}{1- Pe}$$

**Tyrosine hydroxylases immunocytochemistry**

Rats were deeply anesthetized and transcardially perfused with physiological saline followed by 4% paraformaldehyde for fixation. The brains were extracted and immersed in 4% Paraformaldehyde overnight, then trimmed and processed for paraffin embedding. The 3-4 μm thick sagittal brain sections were cut by a rotatory microtome to demonstrate the SNpc and striatum. The brain sections were deparaffinized by xylene and a descending grade of alcohol, followed by washing with phosphate-buffered saline (PBS). Sections were immersed for 30 minutes in 3% H_2_O_2_ to remove endogenous peroxidase activity and incubated for 60 minutes at room temperature (RT) in 10% normal goat serum in PBS and overnight at 4 °C in a Rabbit monoclonal to TH (Abcam, UK). Afterward, the sections were incubated with Goat Anti-Rabbit IgG (Servicebio, China) for 2 hours at RT. Then, we used diaminobenzidine to stain for 5 minutes to visualize the brown color precipitate at the antigen site, followed by hematoxylin to stain slightly. At last, TH positive expression in the SNpc and striatum was observed under the microscope slide scanner SLIDEVIEW VS200 (Olympus, Japan) after being sealed with neutral balsam on slides.

Glossary

| PD | Parkinson’s disease |
| --- | --- |
| ECoG | electrocorticography |
| EMG | electromyogram |
| CMC | corticomuscular coherence |
| SVM | support vector machine |
| SNpc | substantia nigra pars compacta |
| RBD | rapid eye movement sleep behavior disorder |
| EEG | electroencephalogram |
| EOG | electrooculogram |
| WAKE | wakefulness |
| LFPs | local field potentials |
| STN | subthalamic nucleus |
| 6-OHDA | 6-hydroxydopamine |
| M1 | frontal motor cortex |
| REM | rapid eye movement |
| NREM | non-rapid eye movement |
| DBS | deep brain stimulation |
| MFB | medial forebrain bundle |
| FFT | Fast Fourier Transform |
| ANOVA | analysis of variance |
| RBF | radial basis function |
| ROC | receiver operating characteristic |
| AUC | area under the curve |
| TP | true positive |
| TN | true negative |
| FP | false positive |
| FN | false negative |
| P | overall accuracy |
| Pe | expected classification accuracy |
| TH | tyrosine hydroxylase |
| PBS | phosphate-buffered saline |
| RT | room temperature |
| S train | Sham train |
| S test | Sham test |
| L train | Lesion train |
| L test | Lesion test |
| Pre | precision |
| Sen | sensitivity |
| Spe | specificity |
| Acc | overall accuracy |
| Kap | Cohen's kappa |

Table S1. Performance of sleep-wake classifier constructed using occipital ECoG + CMC + EMG in the Sham group

|  | S train – S test | | | S train – L test | | |
| --- | --- | --- | --- | --- | --- | --- |
|  | REM | NREM | WAKE | REM | NREM | WAKE |
|  | | | | | | |
| Precision | 90.95%±7.32% | 90.97%±3.48% | 79.11%±11.65% | 82.86%±28.48% | 82.29%±9.66% | 87.02%±10.27% |
| Sensitivity | 77.94%±7.29% | 93.35%±3.21% | 82.22%±8.66% | 67.19%±16.54% | 96.31%±2.89% | 72.34%±7.89% |
| Specificity | 99.04%±0.72% | 82.98%±4.54% | 94.51%±2.83% | 98.72%±2.06% | 72.10%±9.49% | 96.41%±2.09% |
| Overall accuracy | 88.97%±2.44% | | | 85.41%±4.21% | | |
| Cohen’ s kappa | 0.77±0.05 | | | 0.69±0.07 | | |
|  | | | | | | |

Table S2. Performance of sleep-wake classifier constructed using occipital ECoG + CMC + EMG in the Lesion group.

|  | L train – L test | | | L train – S test | | |
| --- | --- | --- | --- | --- | --- | --- |
|  | REM | NREM | WAKE | REM | NREM | WAKE |
|  | | | | | | |
| Precision | 84.40%±22.29% | 84.52%±9.03% | 82.74%±12.49% | 92.07%±5.74% | 93.78%±2.49% | 68.85%±12.98% |
| Sensitivity | 74.91%±14.73% | 86.48%±3.07% | 75.40%±7.74% | 74.67%±16.54% | 88.13%±2.84% | 89.14%±7.85% |
| Specificity | 99.05%±1.18% | 76.73%±7.88% | 94.94%±2.13% | 99.14%±0.57% | 89.63%±4.55% | 88.78%±2.44% |
| Overall accuracy | 86.29%±3.33% | | | 87.13%±2.94% | | |
| Cohen’ s kappa | 0.71±0.05 | | | 0.75±0.06 | | |
|  | | | | | | |

Table S3. Performance of sleep-wake classifier constructed using frontal ECoG + CMC + EMG in the Sham group.

|  | S train – S test | | | S train – L test | | |
| --- | --- | --- | --- | --- | --- | --- |
|  | REM | NREM | WAKE | REM | NREM | WAKE |
|  | | | | | | |
| Precision | 90.28%±3.91% | 90.15%±3.02% | 80.24%±12.52% | 79.72%±30.98% | 79.38%±10.73% | 88.16%±11.14% |
| Sensitivity | 69.38%±13.21% | 93.26%±2.72% | 84.31%±7.75% | 62.18%±10.46% | 97.41%±2.57% | 62.89%±11.85% |
| Specificity | 98.89%±0.66% | 81.84%±4.01% | 94.50%±2.15% | 98.48%±2.29% | 65.58%±10.04% | 97.36%±1.83% |
| Overall accuracy | 88.49%±2.87% | | | 83.23%±5.34% | | |
| Cohen’ s kappa | 0.76±0.06 | | | 0.64±0.08 | | |
|  | | | | | | |

Table S4. Performance of sleep-wake classifier constructed using frontal ECoG + CMC + EMG in the Lesion group.

|  | L train – L test | | | L train – S test | | |
| --- | --- | --- | --- | --- | --- | --- |
|  | REM | NREM | WAKE | REM | NREM | WAKE |
|  | | | | | | |
| Precision | 79.55%±27.57% | 86.32%±6.72% | 81.59%±14.92% | 82.04%±6.37% | 93.23%±1.86% | 69.60%±14.92% |
| Sensitivity | 70.80%±14.09% | 93.98%±5.89% | 76.30%±11.44% | 74.23%±16.02% | 85.38%±6.57% | 89.29%±4.43% |
| Specificity | 98.39%±2.05% | 78.13%±11.62% | 94.07%±4.25% | 97.49%±1.70% | 88.63%±3.17% | 88.75%±3.89% |
| Overall accuracy | 85.97%±3.58% | | | 85.39%±4.39% | | |
| Cohen’ s kappa | 0.70±0.07 | | | 0.72±0.07 | | |
|  | | | | | | |

Table S5. Performance of PD diagnostic classifier constructed using frontal PD diagnostic features.

|  | Sham | Lesion |
| --- | --- | --- |
|  | | |
| Precision | 95.68%±5.98% | 98.37%±1.41% |
| Sensitivity | 98.30%±1.54% | 95.07%±7.12% |
| Specificity | 95.07%±7.12% | 98.30%±1.54% |
| Overall accuracy | 96.71%±3.07% | |
| Cohen’ s kappa | 0.93±0.06 | |
|  | | |

Table S6. Performance of sleep-wake combined with PD diagnostic classifier constructed using occipital ECoG + CMC + EMG + frontal PD diagnostic features

|  | S-REM | S-NREM | S-WAKE | L-REM | L-NREM | L-WAKE |
| --- | --- | --- | --- | --- | --- | --- |
|  | | | | | | |
| Precision | 88.73%±6.29% | 85.52%±7.42% | 78.39%±11.87% | 79.46%±29.98% | 87.56%±8.62% | 76.69%±14.58% |
| Sensitivity | 75.13%±13.05% | 92.10%±2.40% | 78.98%±9.30% | 30.31%±20.91% | 92.25%±7.31% | 72.31%±16.04% |
| Specificity | 99.50%±0.21% | 92.91%±2.91% | 97.33%±1.47% | 99.85%±0.14% | 93.18%±5.43% | 96.88%±1.67% |
| Overall accuracy | 84.87%±2.22% | | | | | |
| Cohen’ s kappa | 0.79±0.03 | | | | | |
|  | | | | | | |

Table S7. Performance of sleep-wake combined with PD diagnostic classifier constructed using frontal ECoG + CMC + EMG + frontal PD diagnostic features

|  | S-REM | S-NREM | S-WAKE | L-REM | L-NREM | L-WAKE |
| --- | --- | --- | --- | --- | --- | --- |
|  | | | | | | |
| Precision | 82.89%±10.04% | 84.47%±8.22% | 78.49%±11.69% | 60.41%±41.08% | 87.28%±9.10% | 75.75%±14.63% |
| Sensitivity | 74.88%±12.63% | 91.68%±2.86% | 78.52%±9.06% | 24.64%±18.63% | 92.29%±7.85% | 69.89%±16.28% |
| Specificity | 99.19%±0.32% | 92.27%±3.48% | 97.35%±1.41% | 99.81%±0.26% | 92.95%±5.78% | 96.79%±1.66% |
| Overall accuracy | 83.90%±2.50% | | | | | |
| Cohen’ s kappa | 0.78±0.03 | | | | | |
|  | | | | | | |


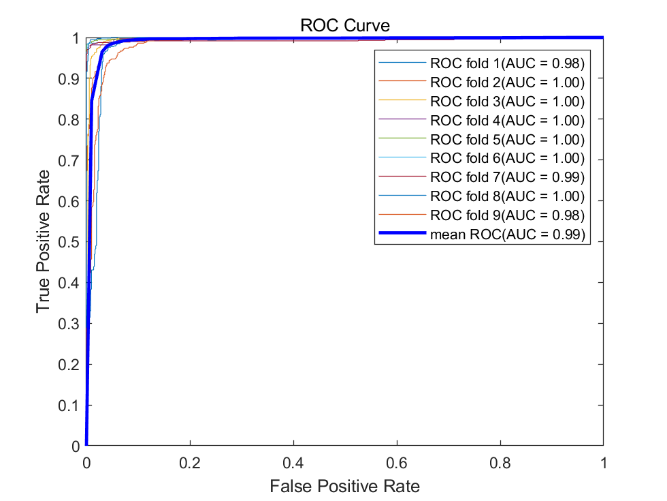


Figure S1. ROC Curve and AUC of PD diagnostic classifier.
